# The Prolyl Isomerase Pin1 Acts Synergistically with CDK2 to Regulate the Basal Activity of Estrogen Receptor α in Breast Cancer

**DOI:** 10.1371/journal.pone.0055355

**Published:** 2013-02-04

**Authors:** Chiara Lucchetti, Isabella Caligiuri, Giuseppe Toffoli, Antonio Giordano, Flavio Rizzolio

**Affiliations:** 1 Sbarro Institute for Cancer Research and Molecular Medicine, Center for Biotechnology, College of Science and Technology, Temple University, Philadelphia, Pennsylvania, United States of America; 2 Human Health Foundation, Terni and Spoleto (PG), Italy; 3 Department of Human Pathology and Oncology, University of Siena, Siena (SI), Italy; 4 Division of Experimental and Clinical Pharmacology, Department of Molecular Biology and Translational Research, National Cancer Institute and Center for Molecular Biomedicine, Aviano (PN), Italy; Karolinska Institutet, Sweden

## Abstract

In hormone receptor-positive breast cancers, most tumors in the early stages of development depend on the activity of the estrogen receptor and its ligand, estradiol. Anti-estrogens, such as tamoxifen, have been used as the first line of therapy for over three decades due to the fact that they elicit cell cycle arrest. Unfortunately, after an initial period, most cells become resistant to hormonal therapy. Peptidylprolyl isomerase 1 (Pin1), a protein overexpressed in many tumor types including breast, has been demonstrated to modulate ERalpha activity and is involved in resistance to hormonal therapy. Here we show a new mechanism through which CDK2 drives an ERalpha-Pin1 interaction under hormone- and growth factor-free conditions. The PI3K/AKT pathway is necessary to activate CDK2, which phosphorylates ERalphaSer294, and mediates the binding between Pin1 and ERalpha. Site-directed mutagenesis demonstrated that ERalphaSer294 is essential for Pin1-ERalpha interaction and modulates ERalpha phosphorylation on Ser118 and Ser167, dimerization and activity. These results open up new drug treatment opportunities for breast cancer patients who are resistant to anti-estrogen therapy.

## Introduction

In the normal mammary gland, estrogen receptor alpha (ERalpha) and its ligand, estradiol (E2), primarily control the ductal outgrowth and side branching that occur during pregnancy and the menstrual cycle [1], [2]. Only 15–25% of mammary epithelial cells express ERalpha. These cells appear to be in a non-proliferative state and act to stimulate the growth of surrounding ERalpha-negative cells in response to estrogen. By contrast, the majority (70%–80%) of primary breast tumors express high levels of ERalpha and the growth of these tumors is estrogen-regulated [3]. As a consequence, inhibition of ERalpha activity by hormonal therapies reduces recurrence and improves clinical outcomes in breast cancer patients [4], [5].

ERalpha belongs to the superfamily of steroid nuclear receptor transcription factors and shares four main functional domains with other nuclear hormone-responsive receptors: the N-terminal transcriptional activation function (AF-1) domain, DNA-binding domain (DBD), hinge region and ligand-binding domain (LBD) (AF-2). When estradiol binds to the AF-2 domain, it induces structural changes that facilitate ERalpha dimerization, nuclear translocation and the binding of DNA to regulate gene transcription [6]. The AF-1 domain is regulated by growth factor receptor signaling, including epidermal growth factor receptor (EGFR), HER2 and insulin-like growth factor receptor (IGF1-R) [7]. The DBD and LBD domains are structurally ordered with a globular profile when expressed independently [8]. The three-dimensional native-fold structure of the N terminal and hinge domains has not yet been resolved as they are intrinsically disordered regions [9].

The hinge region, although initially characterized as merely a flexible connection between the DBD and LBD regions, has recently been discovered to have a more complex function. Indeed, it comprises the nuclear translocation signal and includes estrogen-independent regulatory sequences: selective mutation studies showed that the binding of ERalpha to c-Jun and Sp-1 transcription factors requires an intact hinge domain [10]. The complexity of this domain is further increased since its functional transactivation is mediated by many posttranslational modifications such as methylation, acetylation, sumoylation and phosphorylation [11]–[13]. In particular, the hinge region contains Ser294, a canonical Ser/Thr-Pro motif. This motif has been discovered to have a pivotal function in the regulation of protein activity.

The Ser/Thr-Pro motifs exist in two distinct conformations: *cis* and *trans* [14]. Significantly, phosphorylation on Ser/Thr-Pro motifs has a steric hindrance function that further restrains the already slow cis/trans prolyl isomerization of peptide bonds [15]. Peptidylprolyl isomerase 1 (Pin1) is a unique member of one out of three protein families, the parvulins, which interacts with and isomerizes phosphorylated Ser/Thr-Pro motifs. The final result of the isomerization activity of Pin1 is a conformational change that can alter protein function, localization and/or stability [16]. For the aforementioned reasons, Pin1 has multiple roles in tumorigenesis [17] with important implications in breast cancer development and resistance to hormonal therapy [18]–[22]. With regards to ERalpha, it has recently been reported that Pin1 can directly and indirectly regulate its activity. Pin1 can stimulate the function of ERalpha by promoting SRC-3 coactivator activity and turnover [23] or by promoting CDK2-dependent SMRT corepressor protein degradation [24]. Finally, it has been demonstrated that Pin1 can directly interact with FBS- or estrogen-stimulated ERalpha, regulating its transcriptional activity through Ser118 [4]. ERalphaSer118, Ser167, and to a lesser extent, Ser104/106, are the main residues phosphorylated in the AF-1 domain [25]–[29] and have a central role in the regulation of transcriptional activity of the receptor [25].

In the present study, we show that Pin1 regulates the functions of ERalpha under basal conditions through its interaction with Ser294. We demonstrate that a mutation of Ser294 reduces the levels of Ser118 and Ser167 phosphorylation with important implications for ERalpha dimerization and activation. Finally, we propose a new working model for Pin1 and ERalpha and suggest a novel pathway that can be inhibited to overcome resistance to hormonal therapy.

## Materials and Methods

### Cells culture conditions

MCF7 breast cancer cell lines were purchased from American Type Culture Collection (ATCC, Rochville, MD, USA), 293FT from Invitrogen (Invitrogen Corp, Carlsbad, CA, USA). Cells were grown at 37°C, in a 5% CO2/95% atmosphere. Hormone-free medium was prepared with phenol red–free EMEM with 2 mmol/L L-glutamine, 0.1 mmol/L nonessential amino acids, 50 units/mL penicillin, 50 µg/mL streptomycin, and 3% charcoal-stripped FBS.

### Reagents

Antibodies were purchased from: Pin1 (600-401-A20), 6XHis (600-401-382) from Rockland Immunochemicals, Gilbertsville, PA, USA; ERalpha (sc-8002), ERalphaS-118 (sc-101675), p-AKT (sc-7985R), from Santa Cruz Biotechnology, Santa Cruz, CA, USA;

ERalphaS-167 (31478), from ABCAM Cambridge, MA, USA; α-tubulin (T-6074) from Sigma Inc., St Louis, MO, USA; AKT (9272), MAPK (9102), p-MAPK (9101) from Cell Signaling, Beverly, MA, USA. Kinase inhibitors were purchased from: flavopiridol (F3055) from Sigma Inc., St Louis, MO; AG825 (1555) and U0126 (1144) from Tocris Bioscience, Ellisville, Missouri 63021, USA; LY294002 (9901) from Cell Signaling, Beverly, MA, USA. Estradiol was purchased from Sigma Inc., St Louis, MO, USA

### Plasmids

shRNA plasmids Pin1 (SHCLNG-NM_006221), CDK1 (SHCLNG-NM_001786), CDK2 (SHCLNG-NM_001798) and CDK4 (SHCLNG-NM_000075) were obtained from Sigma Inc., St Louis, MO, USA. Scrambled shRNA (17920), psPAX2 packaging plasmid (12260) and pMDG.2 envelope plasmid (12259) were obtained from Addgene Inc, Cambridge, MA, USA.

For GST pull-down experiments, the IMAGE: 3941595 clone was utilized to amplify the Pin1 human gene with the oligonucleotide primers PIN1-BamHIF GCGGATCCGCGGCAGGAGGGAAGATGG at the 5′ end and PIN1-EcoRIR GCGAATTCCTGGGCTCCCCACCCTCAC at the 3′ end with BamHI and EcoRI adaptor sequences, respectively. The PCR generated products were ligated in the pGEX-2T plasmid for the prokaryotic expression vector (Stratagene Inc., La Jolla CA, USA).

ERalpha- His-AB, His-CD and His-EF plasmids were derived from VP16-ERalpha (ADDGENE:11351) following amplification with primers: AB-BamHI-F ATG GAT CCA CCA TGA CCA TGA CCC TCC-3′, AB-EcoRI-Rev 5′-ATG AAT TCT CCT TGG CAG ATT CCA TAG-3′, CD-BamHI-F 5′-ATG GAT CCA CCA TGG CCA AGG AGA CTC GCT ACT GTG-3′, CD-EcoRI-Rev 5′-ATG AAT TCT TCT TAG AGC GTT TGA TCA TG-3′. EF-BamHI-F 5′-ATG GAT CCA CCA TGT CTA AGA AGA ACA GCC TGG CC-3′, EF-EcoRI-Rev 5′-ATG AAT TCC AGA CCG TGG CAG GGA AAC-3′. After BamHI/EcoRI double digestion, fragments were ligated in pcDNA6 His/Myc vector. ERalpha-His-CDSer294Ala was generated by site-directed mutagenesis with the QuickChange mutagenesis kit (Stratagene, La Jolla, CA, USA).

To obtain pM-GAL4DBD-ERalpha, we digested VP16-ERalpha with EcoRI. The pM destination vector was digested with BamHI and XbaI. The ERalpha fragment and pM vector ends were filled with Klenow polymerase and ligated.

ERalphaSer294Ala was generated by site-directed mutagenesis with the QuickChange mutagenesis kit (Stratagene, La Jolla, CA, USA) and confirmed by direct sequencing. VP16-ERalpha was used as the template for the mutagenesis. The primers used were: ERS294A-f1 5′-GCTGCCAACCTTTGGCCAGCCCCGCTCATGATCAAACGC-3′ and ERS294A-r1 5′-GCGTTTGATCATGAGCGGGGCTGGCCAAAGGTTGGCAGC-3′

All the plasmids were sequence verified.

### Lentiviral production

To generate knock down cells, lentiviral particles were produced as described (http://www.broadinstitute.org/genome_bio/trc/publicProtocols.html) and Rizzolio et al. [30].

### Real-time PCR

Total RNA was prepared from tissues using the RNA extraction kit RNAeasy (Qiagen Inc, Valencia, CA, USA). One µg of total RNA was reverse transcribed in a 20 µl reaction using M-MLV reverse transcriptase (Invitrogen, Carlsbad, CA, USA). Primers to amplify *CTSD, TFF1, GAPDH* were as follows: CTSD-f 5′-GCT GGG AGG CAA AGG CTA CAA-3′ CTSD-r 5′-TCC TGC TCT GGG ACT CTC CT-3′, TFF1-f 5′-CCC TGG TGC TTC TAT CCT AAT A-3′, TFF1-r 5′-AGA AGC GTG TCT GAG GTG TCC-3′, GAPDH-f 5-GAA GGT GAA GGT CGG AGT-3′, GAPDH-r 5-CAT GGG TGG AAT CAT ATT GGA-3′. Quantitative Real-Time PCR (qRT-PCR) was performed with SYBR Green PCR Master Mix (Roche Diagnostic, Basel, Switzerland) in a LightCycler® 480 Real-Time PCR System instrument (Roche Diagnostic, Basel, Switzerland). Samples were run in triplicates and the efficiency of each primer was calculated utilizing an internal standard control [31] [32]. All values were normalized for *GAPDH*.

### GST pull-down assay

GST and GST-Pin1 proteins were produced in BL21 bacteria cells. Cells were grown to mid log phase and then induced to express protein by adding 0.25 mM of isopropyl-1-thio-b-D-galactopyranoside (IPTG, Roche Applied Science, Indianapolis, IN, USA). The cultures were shaken for 4 h; bacteria were then pelleted and resuspended in NENT buffer (20 mM Tris (pH 8), 100 mM NaCl, 1 mM ethylenediaminetetraacetic acid (EDTA), 0.5% NP-40). Cell suspensions were sonicated and pelleted so that the supernatant could be collected. The supernatant was incubated with glutathione agarose beads (Sigma Inc., St Louis, MO, USA) overnight at 4°C. The agarose beads were pelleted and washed three times in NENT buffer. The GST protein was analyzed by electrophoresis gel and blue coomassie staining. 1 mg of protein was pulled down with 10 ug of GST or GST-Pin1.

### Co-immunoprecipitation assay

Sub-confluent MCF7 cells were harvested and proteins were prepared as follows: the cell pellet was resuspended in lysis buffer (20 mM Tris HCl pH 8, 137 mM NaCl, 10% glycerol, 1% NP40, 2 mM EDTA). 1 mg of proteins was immunoprecipitated, utilizing 4 µg of Pin1, ERalpha antibody or mouse IgG overnight at 4°C. The immunoprecipitated protein complex was collected with agarose protein A/G beads (Pierce) for 3 h at 4°C. After washing, the protein immunocomplex was run on SDS-PAGE followed by immunoblot analyses to detect Pin1 or ERalpha proteins.

### In vitro kinase assays

GST-D WT or mutant constructs were purified from E. Coli, 1 µg of recombinant protein incubated with 100 U of CDK2/CycA2, CDK4/CycD1, CDK6/CycD1 (SignalChem, Richmond, Canada) in the kinase reaction buffer (40 mM Tris HCl, 20 mM MgCl2, 0.1 mg/ml BSA, 0.2 mM, ATP, 2 mM DTT) for 30 minutes at RT. The reaction was analyzed with the Kinase Glo luminescence assay (Promega, Wisconsin, USA) according to the manufacturer's protocol.

### Dimerization assay

293FT cells were seeded in 96 multiplate wells at 2×10^4^ cells/well and grown in hormone-free medium. After three days, cells were transfected (Fugene HD, Roche Applied Science, Indianapolis, IN, USA) with 300 ng of VP16ERalpha and pM-GAL4DBD-ERalpha wild-type or Ser294Ala mutant, 300 ng of GAL4-LUC-pGL2Basic (Promega E1641) and 50 ng of renilla plasmid (Promega, E2241). After 24 hours, cells were treated with 10 nM of 17-β-estradiol for 12 hours. The luminescence of each sample was measured in a single tube luminometer (Berthold Technologies, GmbH & CO, Germany) with the Dual-Luciferase Reporter Assay System (Promega, E1910). Each transfection was performed three times in order to overcome the variability inherent in the experiment.

### Statistical analysis

Statistical analyses were performed using GraphPad software by applying unpaired Student's *t*-test. Quantification of western blot was carried out with Adobe Photoshop.

## Results

### Pin1 interacts with ERalpha under basal conditions

**Figure 1:**
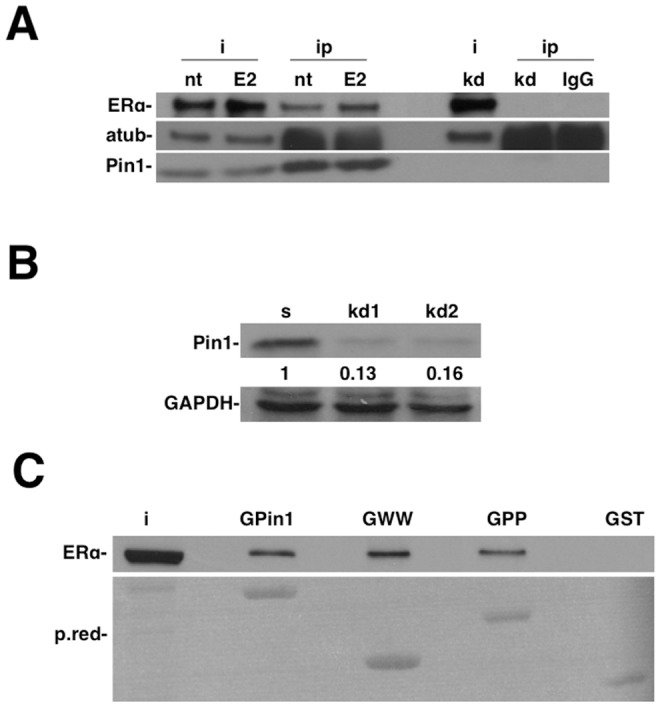
Pin1 associates with ERalpha both *in vitro* and *in vivo*. A) Serum-starved cells were treated with estradiol (E2) or ethanol for 45′. Cells were harvested and pellets immunoprecipitated with anti-Pin1, and analyzed by Western blot with anti-ERalpha antibody. Pin1 kd cells or IgG were used as a negative control. B) MCF7 cells were transfected with shRNA scramble (SCR) or shRNA Pin1 (kd1 and kd2), and Pin1 protein level was detected by immunoblotting. GAPDH was used as the load control. Quantification represents the ratio between the Pin1 and GAPDH proteins and normalized to scrambled cells. C) GST, GST-Pin1, GST-WW or GST-PPIase fusion proteins were incubated with ERalpha-positive MCF7 cell lysates and the bound proteins were analyzed by immunoblotting with an ERalpha antibody.

Previous studies demonstrated that Pin1 indirectly controls ERalpha activity [23], [24] or via direct binding to phosphorylated Ser118 under hormone or growth factor stimulation [4]. To further clarify the role of Pin1 in the regulation of ERalpha activity, we immunoprecipitated Pin1 in the presence or absence of 17-β-estradiol in MCF7 cells (Fig. 1A). Pin1 kd cells (Fig. 1B) and IgG were used as a negative control. We demonstrated that, under both conditions, Pin1 forms a macromolecular complex with ERalpha *in vivo*. To understand which mechanism is involved in the interaction between Pin1 and ERalpha under basal conditions, we then decided to carry out all experiments in the absence of FBS and estradiol unless indicated.

Pin1 is a short protein that contains two known functional domains, a WW interacting domain (amino acids 6–37) and a PPIase domain (amino acids 54–163). Total cellular lysate was incubated with GST, GST-Pin1 protein, GST-WW and GST-PP domains (Fig. 1C). GST pull-down assays confirmed that both the WW domain and PPIase domain are critical for ERalpha interaction.

### The CD domain in ERalpha is responsible for Pin1 binding

**Figure 2:**
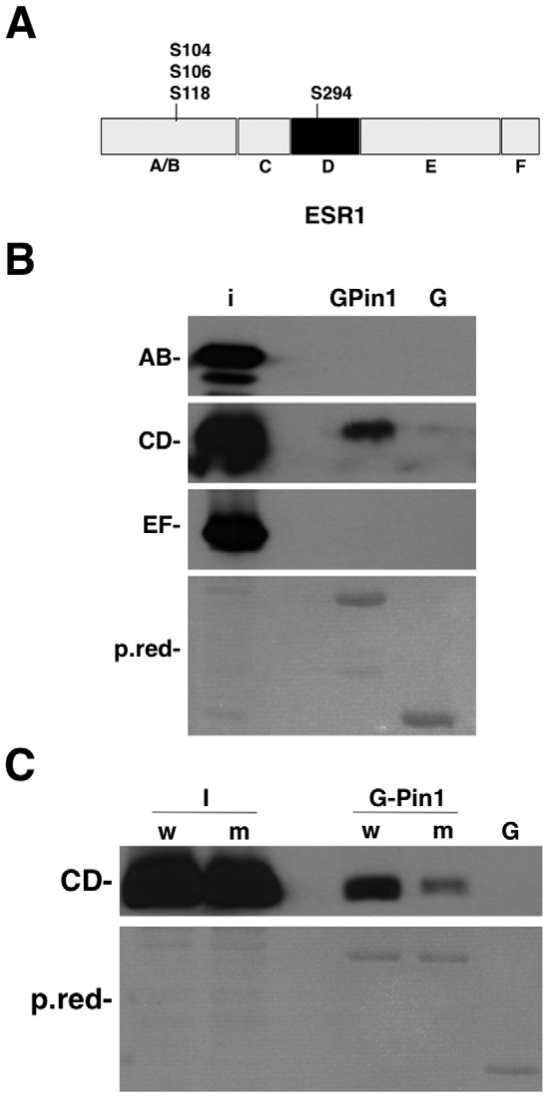
ERalpha CD domain interacts with Pin1. A) Schematic representation of ERalpha domains, showing the predicted prolyl isomerase sites (Ser/Thr-Pro). B) ERalpha cDNA was split into three different domains (AB, CD, EF) and tagged with histidine. GST or GST-Pin1 pull-down assay was performed in 293FT cells and analyzed by Western blot with anti-6×His antibody. C) Site-directed mutagenesis was performed to replace Serine 294 with Alanine on the ERalphaCD domain. Plasmids with wild-type (w) or mutant (m) CD His-tagged domain were transfected in 293FT cells and pulled down with GST-Pin1 protein. Anti-His antibody was used in Western blots to detect the interaction.

To clarify which ERalpha domain is involved in the binding of Pin1, ERalpha protein was split into three regions (domains A/B, CD and EF) (Fig. 2A) and fused with a C-terminal Histidine tag for further analysis. Plasmids were transfected in 293FT cells and total cellular lysate was pulled down with GST and GST-Pin1 proteins (Fig. 2B). Using a 6×-His antibody, we were able to demonstrate that only CD peptide interacts with GST-Pin1 but not GST. As Pin1 is a peptidyl-prolyl-isomerase that specifically recognizes and isomerizes phosphorylated Ser/Thr-Pro motifs, we explored putative sites in the CD region that match the consensus motif. We found that Ser294 was a unique Ser-Pro site located in the D domain (Fig. 2A). To confirm that Ser294 is responsible for the binding between ERalpha and Pin1, a Ser294Ala mutation (MT) was introduced by site-directed mutagenesis. 293FT cells were transfected with CD wild-type and mutant domains. GST-pull down experiments showed that mutation in the CD peptide greatly reduced the ability of the ERalpha protein to bind to Pin1, (Fig. 2C) confirming Ser294 as the Pin1 binding site.

### CDK2 interacts with and phosphorylates the CD peptide to generate the Pin1-binding site

**Figure 3:**
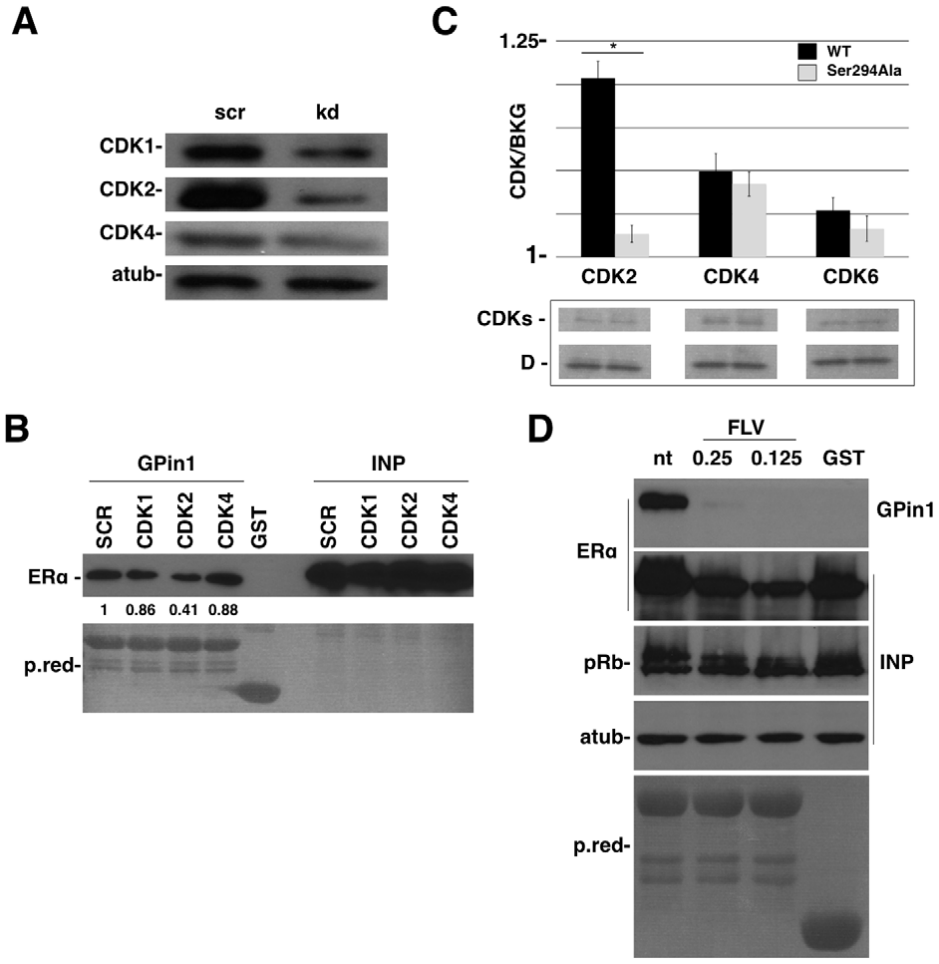
CDK2 allows ERalphaSer294 and Pin1 interaction. A) 293FT cells were infected with 1 MOI of specific CDKs or Scrambled shRNA lentivirus as indicated. B) 293FT cells knocked down for CDK1, CDK2 and CDK4 genes were transfected with ERalphaCD domain and pulled down with GST-Pin1. Quantification represents the ratio between the pull down and total ERalpha protein and normalized to scrambled cells. C) Kinase assay was done on wild-type or mutant ERalphaD domains. The D domain was incubated with activated CDK/cyclin complexes as indicated and the kinase activity was measured in Relative Luciferase Unit/Second (RLU/s). Data shown represent average values ± S.D. *n* = 3. In the lower panel, the GST-D domain and cyclin/CDKs complexes loading controls. D) Cells were treated with flavopiridol at different drug concentrations and pulled down with GST or GST-Pin1. The level of phosphorylation of pRb was used to control the efficacy of flavopiridol.

Since Pin1 activity requires prior phosphorylation of its target site and the Ser/Thr-Pro motif is the minimal consensus sequence recognized by Cyclin Dependent Kinases (CDKs) [33], we investigated whether CDK-mediated phosphorylation of Ser294 elicits Pin1 binding. For this purpose, shRNA CDK1, CDK2 and CDK4 were stably expressed in 293FT cells (Fig. 3A) and transfected with the ERalpha CD domain. GST-Pin1 was used as a bait protein to immunoprecipitate the CD domain. After pull down, we observed a reduced amount of the CD domain in 293FT cells expressing shRNA CDK2 compared to shRNA CDK1 or CDK4 proteins (Fig. 3B). To confirm this result, a kinase assay was performed on WT and Ser294Ala D domains and used as a substrate for the active form of CDK2/CycA, CDK4/CycD1 and CDK6/CycD1 kinase complexes. Detection of kinase activity was performed using Kinase Glo luminescence assay. CDK2 efficiently phosphorylates the wild-type D domain and to a lesser extent the Ser294Ala D domain. CDK4 and CDK6 show a very weak activity on the D domain. The result confirms that CDK2 can bind and phosphorylate ERalpha in the D domain and facilitates Pin1 binding by generating a phospho-Ser-Pro site (Fig. 3C).

In order to further demonstrate the involvement of the CDK pathway in Pin1-ERalpha binding, we decided to use flavopiridol (FLV), a potent CDKs-inhibitor. MCF7 cells cultured in phenol-red-free and hormone-free medium were treated for 16 hours with 0.25-0.125 µM concentrations of FLV. As expected, inhibition of CDK activity blocked Pin1 binding on ERalpha (Fig. 3D).

### Ser294 is involved in Ser118 and Ser167 phosphorylation and participates in ERalpha dimerization

**Figure 4:**
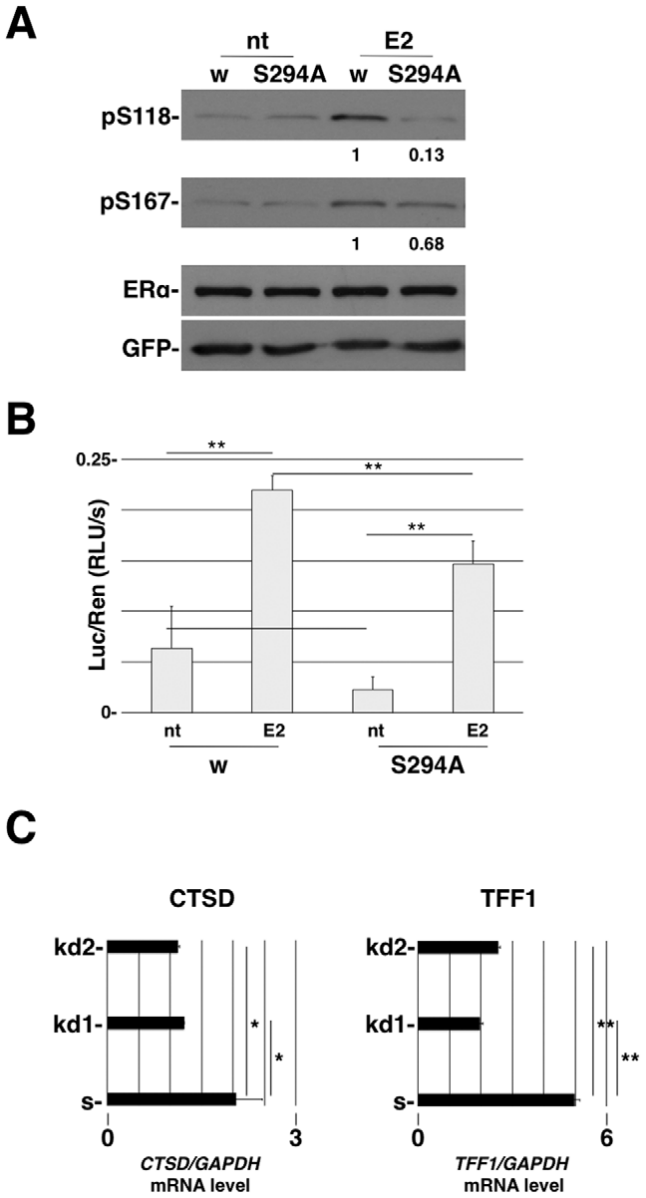
Ser294 is involved in Ser118 and Ser167 phosphorylation and participates in ERalpha dimerization. A) 293FT cells were transfected with ERalpha wild-type (W) and Ser294Ala plasmid constructs. After 17-β-estradiol treatment, cell lysates were immunoblotted and probed with ERalpha Ser118 and Ser167 phospho-specific antibodies. The GFP plasmid was utilized as the internal control of transfection. Quantification represents the ratio between the phosphorylated and total ERalpha protein and normalized to WT ERalpha protein. B) The pGL2 Luciferase reporter construct was transfected into 293FT cells together with either GAL4/VP16 ERalpha WT or GAL4/VP16 ERalpha Ser294Ala plasmids. The values represent the luciferase activity normalized to Renilla. Transfected cells were treated with 10 nM 17-β-estradiol to stimulate ERalpha dimerization. The data represent the average of three different experiments. Error bars represent ±SD. p-value<** 0.01 C) Real-time PCR analysis of CTSD and TFF1 ERalpha-target genes in scrambled and Pin1 kd cells. The values are normalized to the GAPDH gene. Data represent the average of three replicates. Error bars represent ±SD. p-value<* 0.05 or ** 0.01.

Phosphorylation of ERalpha on serine 118 and 167 is required for the full activity of protein function [25]. Both residues are located within the N-terminal region (AF-1), which is known to promote transcription in a ligand-independent manner. However, phosphorylation of these serines is enhanced in response to ligand binding in the AF-2 domain. In order to examine the significance of Ser294 in relation to ERalpha phosphorylation *in vivo*, a full-length ERalpha expression plasmid bearing the mutation Ser294Ala was generated. 293FT cells were co-transfected with ERalpha WT or Ser294Ala and GFP plasmid as controls. Two days before treatment, cells were grown in phenol-red-free medium containing 3% charcoal-stripped FBS. After 45 min of E2 treatment, cellular pellets were collected and lysed for Western blot analysis. We could note that phosphorylation on Ser118 and Ser167, both implicated in ERalpha transcriptional activation, was reduced in cells transfected with the Ser294Ala ERalpha plasmid. The Ser294Ala is located in the D domain, a flexible region that lies between two distinct dimerization interfaces in the DBD and LBD regions [34]. To investigate the involvement of Ser294 in E2-dependent ERalpha dimerization and transcriptional activation, a mammalian two-hybrid assay was used. ERalpha- WT and Ser294Ala constructs were cloned in pGAL4 (DBD) and pVP16 to generate hybrid fusion proteins and transfected as homodimers in 293FT cells. Transfected cells were treated with E2 or a vehicle for 16 h. We observed a statistically significant reduction of ERalphaSer294Ala activity compared to wild-type ERalpha (Fig. 4B). These experiments show that phosphorylation of Ser294 is required for proper phosphorylation of ERalpha following hormone-induced stimulation.

To confirm that Pin1 modifies ERalpha activity, we carried out a real-time PCR (qRT-PCR) on two well-known estrogen-induced genes (CTSD and TFF1) in Pin1 kd MCF7 cells treated with E2 for 6 h (Fig. 4c). We were able to detect a reduction in mRNA levels for both the CTSD and TFF1 genes.

### PI3K/AKT is involved in the Pin1-ERalpha interaction

**Figure 5:**
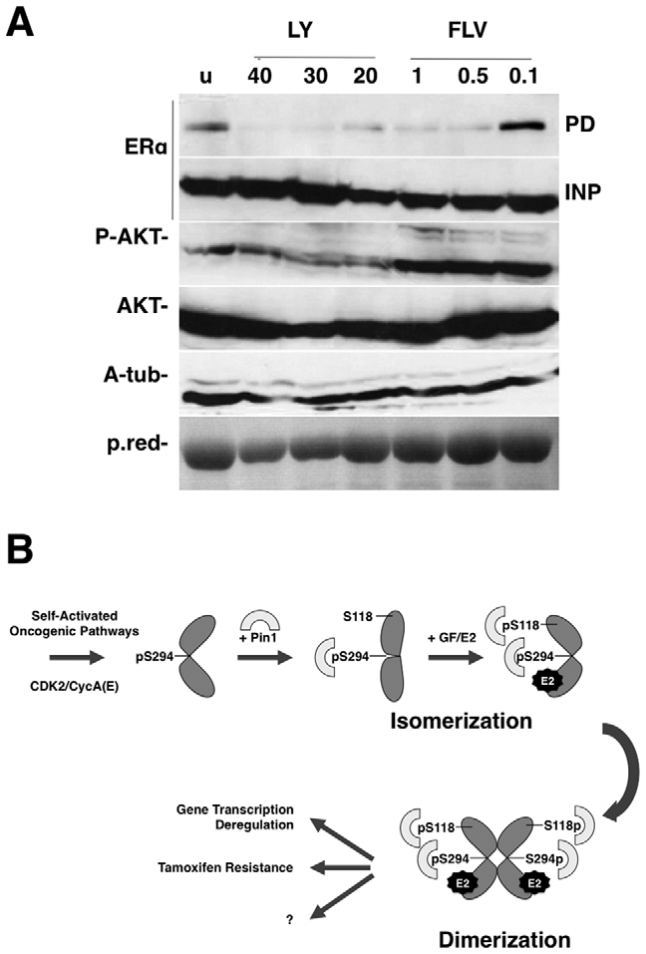
The PI3K-CDK pathway is responsible for interaction between Pin1 and ERalpha. A) MCF7 cells were grown in normal serum and treated with different micro Molar concentration of LY294002 or flavopiridol drugs for 16 hours. Total protein lysates were pulled down (PD) with GST-Pin1. AKT was used as the control of LY294002 treatment. B) Pin1 and ERalpha working model under basal and estradiol-stimulated conditions. See text for details.

ERalpha is regulated by multiple signaling pathways including the PI3K/AKT pathway and the extracellular-regulated kinase pathway (MAPK). Neither pathway excludes the other, and they can activate ERalpha in a ligand-dependent or -independent manner [25]. To investigate which pathways stimulate the Pin1-ERalpha interaction, we treated the MCF7 cells with drug inhibitors of PI3K (LY294002), Her2 (AG825) and Mek1/2 (U0126). We performed the experiments in the absence or presence of FBS, with similar results. GST-pull down with Pin1 fusion protein performed on MCF7 cellular lysates showed a marked reduction in Pin1/ERalpha binding in the presence of the PI3K/AKT inhibitor LY294002 (Fig. 5A and data not shown). These results suggest a contribution of the PI3K/AKT pathway and CDK2 activity on EralphaSer294. Differently, Her2 and Mek1/2 inhibitors did not affect Pin1 binding, excluding their involvement in this mechanism (data not shown).

## Discussion

Evaluation of the ERalpha status is an essential component in the pathological classification of breast cancers and determines the success of endocrine therapies [35]. Endocrine agents are currently used as first line therapy for ERalpha-positive breast cancers [36]. These treatments target the ERalpha pathway by blocking the receptor's activity or starving the tumor of estrogens.

A number of studies suggest that the benefit of endocrine therapy is proportional to the level of ERalpha expression [37]–[39] and therefore further characterization of the factors that determine the precise levels of ERalpha is essential. However, although beneficial, resistance to hormonal therapy occurs in 50% of patients and it remains crucial to determine the underlying molecular mechanism [40]. Recent data support the concept that Pin1 is implicated in the acquired resistance to tamoxifen [4], [18], [41], a drug that has been used in the clinical setting for more than 30 years [7]. In particular, Pin1 is involved in the overexpression of the HER-2 receptor [18] and controls the activity of ERalpha by regulating the stability of its cofactors, SRC-3 [23] and SMRT [24]. Furthermore, it has been demonstrated that under FBS or estradiol conditions, Pin1 can regulate ERalpha activity through interaction with phosphoSer118 [4]. The authors suggested that when Ser118 is phosphorylated, the ERalpha N-terminus is preferentially restricted in the *cis* conformation. After Pin1 binding, the AF1 domain is switched to the *trans* conformation by promoting a slight local structural change [4].

Here we report that under basal conditions, ERalpha Ser294 is the target of Pin1. We demonstrated by GST pulldown and coimmunoprecipitation assays on MCF7 cell extracts that Pin1 interacts with ERalpha and this interaction was reduced when serine 294 was mutated to alanine.

The hinge region is a relatively short domain spanning 51 amino acids (263–314), and with several important functions in ERalpha regulation. Complete deletion of the hinge region impairs ligand-induced downregulation of the receptor [10], [42]. In the N-terminal region (253–282 aa) overlapping the DNA binding domain resides the ERalpha nuclear localization sequence (NLS) that includes a protein-protein interaction sequence (267–275 aa) for c-Jun transcriptional factor [10]. Moreover, sumoylation of lysines 302/303 promotes ligand-induced polyubiquitination, which is required for receptor turnover [43]. The localization of Ser294 in the hinge region near the DBD domain suggests a role [13], [44] in DNA binding and/or dimerization of ERalpha. Using a two-hybrid assay, we demonstrated that Ser294 is effectively involved in dimerization and consequently in ERalpha activation. After estradiol stimulation, a significant reduction in transcriptional activity was detected in cells transfected with ERalpha mutant, suggesting that a Ser294Ala mutation impairs dimerization.

On the basis of results in the literature [4] and our own data [45], we proposed a working model in which Pin1 regulates ERalpha in two different steps: under basal conditions, there is a first event mediated by CDK2 phosphorylation that takes place on Ser294. This step is required for Pin1 binding and subsequent phosphorylation of Ser118 and Ser167. In the second step, growth factor stimulation promotes Pin1 recruitment to ERalpha at phosphoSer118, modulating AF-1 functional activity (Fig. 5B). Since the proper phosphorylation levels of Ser118 and Ser167 have been associated with a clinical response of patients treated with tamoxifen [46]–[48], it appears that Pin1 has a critical role in this mechanism.

Although Ser294 has a proven role in the activity of ERalpha, there is a lack of information regarding the pathway involved in Ser294 phosphorylation and its functional significance. Serines residues located in the N-terminal domain (Ser104/106, Ser118, Ser167) and in the Hinge region (Ser294) match the consensus sequence recognized by families of Serine/Threonine proline directed kinases, including cyclin-dependent kinases. ERalpha transcriptional activity is virtually abolished under conditions where CDK2 activity is suppressed by the kinase inhibitor p27 or by a dominant negative CDK2 mutant, regardless the presence or absence of hormones [49]. Thus it appears that CDK2 is a limiting cofactor in the regulation of ERalpha-dependent transcription. We demonstrated that Ser294 amino acid represents a new target for CDK2, and its phosphorylation is required for Pin1 binding, a mechanism elicited by the PI3K pathway. Currently, the detailed signaling pathway that sustains the Pin1-ERalpha interaction remains obscure although there are different experimental models, which have demonstrated a link between PI3K and CDK2 activity in a different tissue context. The PI3K pathway promotes Thr-160 phosphorylation in the CDK2 activating site [50]–[52]. Of note, Pin1 can stabilize the AKT protein [52], suggesting a multiple level mechanism in which Pin1 controls the PI3K/AKT pathway [53]. Further studies will be done to clarify the PI3K signaling cascade in CDK2/ERalpha regulation in breast cancer.

Overall, our results suggest a new pathway that involves PI3K/AKT, CDK2 and Pin1 and open up new opportunities for treating ERalpha-positive breast cancer patients who are resistant to hormonal therapy.
